# Effects of ambient temperature and humidity on kidney stone admissions in Brazil

**DOI:** 10.1590/2175-8239-JBN-2020-0062

**Published:** 2020-06-01

**Authors:** Sitalakshmi J. Iyer, David S. Goldfarb

**Affiliations:** 1 NYU Langone Medical Center Nephrology Section, New York Harbor VA Healthcare System and Nephrology Division, New York, USA.

Nephrolithiasis is a common and painful urologic condition that poses a significant global disease burden. Its overall prevalence in the US has increased over the last several decades (10.1% in 2014) and investigators from around the world have made similar observations. Several hypotheses for the phenomenon have been proposed including rising prevalence of diabetes, gout, obesity, changes in diet, climate changes, geographic variation, and work hazards.^1^

Geographic variation had first been investigated by Soucie *et al.,* by analyzing data from two cross-sectional surveys (second Cancer Prevention Survey 1982 and National Health and Nutrition Examination Survey 1976-1980) on self-reported, physician-diagnosed kidney stones.^2^^,^ 3 Stone prevalence was stratified by latitude and region with a notable increase from west to east longitude, and more pronounced form north to south latitude in the US.^2^ Whether those observations were the result of heat, sunlight exposure, diet, or genetics could not be established.

In this issue of the Brazilian Journal of Nephrology, Abreu-Jr and Ferreira Filho test the influence of climate on the number of hospitalizations for nephrolithiasis.^4^ Climate refers to the average daily weather for an extended period of time at a certain location, while weather reflects the short-term conditions of the atmosphere at that location. A strength of this study is that, as the authors say, Brazil is “a country of continental proportions”, with “broad variation in temperature between the north (tropical area) and the south (subtropical area)”. Three zones and 12 climates have been classified throughout Brazil and selected cities were chosen from the tropical and sub-tropical regions.

The selected cities in the study under review span a wide latitude range, which is unique and would not be possible in most countries. However, the study included hospitalizations only, but did not include emergency room visits or outpatient urological procedures such as extracorporeal shockwave lithotripsy or other same-day urological procedures. Broader inclusion of stone events would have provided a larger sample cohort leading to lower variability and bias. However, we agree that hospitalizations are probably representative of all kidney stone presentations. They used the minimum, maximum, and mean temperatures, and relative air humidity of each city recorded monthly, reported in degrees Celsius and percentage, respectively. The mean temperature was computed as an arithmetic average of the lowest and highest temperatures, which may not depict a 12 or 24-hour average given the high temperature variability. The authors used a multiple linear regression model, whereas nonlinear models (as used by Ross, see below) might have been more appropriate and caused less discrepancy. To summarize their result, they postulated approximately 500 new hospitalizations per month and more than 7000 new kidney stone hospitalizations per year for an increase of 1ºC in the monthly mean temperature. In addition, a negative association was found between the number of hospitalizations for nephrolithiasis and the relative humidity.

These data are confirmatory of other observations regarding heat and humidity and significantly enhance the available data. However, lacking data on urine chemistry or urine volume, the findings do not lead us to elucidate the mechanisms by which these effects manifest. Increased ambient temperature is a recognized risk factor for stone formation, which has usually been explained as related to heat-associated sweating with a compensatory reduction in urine volume and an increase in urinary supersaturation with stone forming-salts. However, some studies have not confirmed lower urine volume during seasons of hotter temperature and instead report increases in urine calcium excretion as the apparent risk factor.^5^

Temperature variations and metrics have been studied by many researchers. Tasian *et al*. evaluated the exposure-response and lag-response relationships between daily temperature and kidney stones. They found that as daily temperatures increased above 10ºC, the risk of kidney stone presentation over the next 20 days also increased in most US cities.^6^ The lag between high daily temperatures and observed increase in kidney stones was surprisingly short: only 3 days. The authors then used a time series design with distributed lag non-linear models to identify the most appropriate temperature metric for predicting kidney stone presentations in the state of South Carolina over a period of 19 years.^7^ The moist temperature metrics (wet-bulb temperatures and heat index) were better at predicting kidney stone presentations than dry-bulb temperature metrics, particularly in summer, regardless of type of adjustment for relative humidity. We assume that the current study utilized dry-bulb temperature measurements which may affect the pertinence of findings.

In the current study, the authors observed a male/female ratio of 0.9 in tropical climates and 1.1 in subtropical climates, a difference that is probably not statistically different. These data are, however, confirmatory of other observations demonstrating that what was once considered a disorder affecting more men, has progressively affected women in nearly equal proportions. The effects of temperature here are different than we previously demonstrated in the second Cancer Prevention Survey dataset in the US, where the effect of warmer temperatures in increasing kidney stone prevalence was greater in men than in women.^8^ Those data were gathered in 1988. Since then, an increasing prevalence has been seen in women, an effect which we recently reviewed.^9^ We speculate that increased rates of overweight and diabetes, and occupational issues have affected women disproportionately in recent years.

This study’s observations were of urban populations. We recently hypothesized that urban populations are exposed to higher ambient temperatures than rural ones, as the result of urban heat islands.^10^ The world-wide migration of people from rural to urban settings could be an additional cause of the recently described increase in kidney stone prevalence.

Overall, the study has strongly supported the evidence we have about the influence of climate on the incidence of nephrolithiasis. It has limitations: the analysis did not use nonlinear models, the study was retrospective, used an average temperature for analysis, and did not consider dietary or other influences on the risk of kidney stones. Nonetheless, it provides important confirmatory evidence of heat-related nephrolithiasis, which should have implications for the continued health-related effects of climate change.
